# Stabilizing time and its predictors among 1–59 months old children managed for severe acute malnutrition during the humanitarian crisis in Tigray regional state of Ethiopia, 2023: a prospective cohort study

**DOI:** 10.1186/s12887-024-04711-4

**Published:** 2024-04-01

**Authors:** Wagnew Tesfay, Mebrahtu Abay, Berhane Fseha Teklehaimanot, Ataklti Gebremedhin

**Affiliations:** 1 Medical Teams International (MTI), Shire Field Office, Nutrition Supervisor, Shire, Ethiopia; 2 College of Health Sciences, Aksum University, Aksum, Ethiopia; 3 College of Health Sciences, Adigrat University, Adigrat, Ethiopia; 4 Paediatrician in Suhul General Hospital, Shire, Ethiopia

**Keywords:** Severe acute malnutrition, Stabilizing time, Emergency, Tigray, Ethiopia

## Abstract

**Background:**

Higher rate of acute malnutrition is observed in emergencies compared to non-emergency settings and severe acute malnutrition upsurges alarmingly and become deadly in humanitarian crises due to lack of food, lack of quality water supply and insufficient healthcare. Research is one learning tool by identifying strength and areas of improvement. However, little is known about outcomes of therapeutic feeding programmes in comparison with the standard indicators set in humanitarian setting.

**Methods:**

Health facility based prospective cohort study was conducted using routinely collected programme data of children hospitalized to the inpatient therapeutic feeding center in suhul general hospital from January 1st, 2023 to June 30, 2023. Data was collected using a form developed relating to the federal ministry of health standard management protocols for severe acute malnutrition then it was cleaned, coded and entered to EpiData version 4.2.0 and then exported to SPSS version 25 for analysis.

**Results:**

From 184 children, 96.2% were stabilized while the remaining 3.8% were censored with overall median stabilizing time of 8 days. Weight gain was used as one of the discharging criteria for infants less than six months and their mean weight gain found to be 12.89 g per kilogram daily. Appetite test (AHR = 0.338; 95% CI: 0.221–0.518), blood transfusion (AHR = 5.825; 95% CI: 2.568–13.211), IV fluid resuscitation (AHR = 2.017; 95% CI: 1.094–3.717), IV antibiotics (AHR = 2.288; 95% CI: 1.164-4.500) and NG tube feeding (AHR = 1.485; 95% CI: 1.065–2.071) were identified as significant predictors of stabilizing time.

**Conclusion:**

All the outcome indicators for stabilization center are consistent with the SPHERE association set of standards during humanitarian intervention. The hospital and other concerned humanitarian organizations should focus on sustaining these achievements as suhul hospital is the main treatment center for children suffering from severe acute malnutrition in the northwest zone of Tigray regional state. Further pre-post experimental studies which compare the stabilizing time before and after crisis are recommended.

## Background

Humanitarian emergency is broadly defined as any situation where humanitarian needs exceed the managing capacity of a society or government, requiring a wide range of national and international humanitarian actors to respond [1]. A higher rate of acute malnutrition is observed in emergencies compared to non-emergency settings. The world health organization (WHO) describes severe acute malnutrition (SAM) by the presence of severe wasting [mid-upper arm circumference (MUAC) of less than 11.5 centimetre in children 6 to 59 months old, and/or weight-for-length/height (WFL/H) below − 3 Z scores in kids 0–59 months of age); Or bilateral pitting oedema [2].

Acute malnutrition affects tens of millions of children under five years old globally and about 14 million are having SAM. Around 69 and 27 per cent of these children with SAM live in Asia and Africa respectively [3–6]. It still remains global public health concern by underlying about 50% of the estimated 10 to 11 million under five deaths. Severely malnourished children have nine fold immediate risk of death compared to their well-nourished counter parts [7–9]. SAM upsurges alarmingly and become deadly in humanitarian crises due to lack of food, lack of quality water supply and insufficient healthcare [10]. Even though all population groups suffer from SAM during emergencies, it is main cause of morbidity and mortality in infants and young children, predominantly in sub-Saharan Africa and South-East Asia [11].

The recent Ethiopian mini demographic health survey (EMDHS) found that 7% of under five children are wasted, out them the 1% are severely wasted [12]. As Ethiopia is prone to and currently facing humanitarian crises due to conflict and internal displacements this figure is extremely likely to increase, causing unacceptably high under five mortality rates [13, 14]. As a result, the war caused shocking escalation of house hold food insecurity from 41 to 85%, fivefold increase in SAM among children under five and more than seventy per cent non-functionality of the health facilities in the region [15–17]. From the recent nutrition mass screening and measles supplementary immunization activities campaign conducted in the zone in March 2023, Tigray regional health bureau and the United Nations children’s emergency fund (UNICEF) reported (not published) that the overall global acute malnutrition (GAM) rate for under five children was 16.1% while the SAM rate found to be 3.3 per cent [18].

Recent researches on in-hospital mortality among children with SAM in South Africa [9] and Uganda [19] found unacceptably high death rates. Similar cohort studies in higher teaching institution in Ethiopia concluded that the rates of recovery are below the standard although all stated the stabilizing time or length of stay (LoS) is in the acceptable range set by the SPHERE association [20–23]. Conversely, other researches declared that all their findings met the standards [24–26]. Breast feeding, receiving antimicrobials and vaccination increased the rate of stabilization with shorter duration whereas comorbidities like tuberculosis (TB), human immune deficient virus (HIV), diarrheal diseases, pneumonia and anemia lower the chance of recovery [9, 20, 24–28].

Receiving intravenous (IV) fluid and blood transfusion [19, 21], having skin dermatoses as well as altered body temperature at admission [23] and feeding with the help of nasogastric (NG) tube [8, 29] were determinants of stabilizing time in SC. Type of SAM during hospitalization was an independent predictor of stabilization [22, 30]. In an attempt to end all forms of under nutrition and to improve quality of care for hospitalized severely malnourished children, Ethiopia has approved and is implementing national and global commitments, including the WHO endorsed national guideline. Nevertheless, the outcomes of stabilization centers (SC) in sub-Saharan Africa are below the international set of standards [31], while an extended stay in SC is associated with a higher risk of hospital acquired infection that can lead to an increased risk of death [25].

In the humanitarian context, research is one learning tool by identifying strength and areas of improvement. Service quality indicators like stabilizing time and other related outcomes of nutrition interventions during humanitarian crises should be monitored to get better results of efforts and to take further actions evidence-based [32]. However, little is known about outcomes of therapeutic feeding programmes (TFP) in comparison with the standard indicators set in humanitarian setting [6]. Moreover, as universities in the region were not able to conduct researches after the conflict, this study could be the first of its kind in about three years and academia can use it as a reference. Therefore, the aim of this study is to determine the median stabilizing time and its predictors among 1–59 months children admitted to stabilization centre of Suhul General Hospital, Tigray, Northern Ethiopia.

## Methods and materials

### Study design

Hospital-based prospective cohort study was conducted using routinely collected program data of children hospitalized to stabilization centre of Suhul Hospital, Tigray, Ethiopia.

### Context and period of study

Shire town is the zonal capital of northern west Tigray regional state, located 1087 km north of Addis Ababa. As most of the health institutions have been destroyed and the few health facilities sustained are not functioning properly, the inpatient therapeutic feeding center (ITFC) in Suhul hospital has been the main treatment center for children suffering from complicated SAM, both for host and internally displaced persons (IDPs) in north west zone of Tigray. Like almost all healthcare facilities in the region, this hospital has experienced extensive looting of equipment, medical supplies, electrical installations and destruction of water supply system.

Following the cessation of hostilities agreement (CoHA) between the armed combatants in November the 2nd, 2022, humanitarian actors got comparative access to the region and Suhul hospital in particular. As a result, the paediatrics ward of suhul hospital has been receiving supplies and technical support from United Nations’ agencies as well as other international non-governmental organizations (NGOs). Admission, treatment and discharge of children suffering from acute undernutrition were as per the Ethiopian federal ministry of health (FMoH) protocol for the management of SAM [14]. Pediatrician and general practitioners did routine ward rounds to diagnose, prescribe medications and other related decisions while the nutrition nurses were doing appetite test to move children from Phase-I to transition phase and phase-II. Infant and young child feeding (IYCF) counseling was given for mothers or care givers about the situation of their child, sanitation and hygiene practices and the required follow up till recovered. The research was done from January 1st, 2023 to June 30, 2023.

### Source and study population

#### Source population

All 1–59 months old children hospitalized to the SC in suhul general hospital, both from IDP and host communities.

#### Study population

All 1–59 months old children hospitalized to the SC in suhul general hospital, both from IDP and host communities from January 01, 2023 to June 30, 2023.

#### Study unit

Severely malnourished under five child admitted to suhul general hospital SC from January 01, 2023 to June 30, 2023.

### Inclusion and exclusion criteria

#### Inclusion criteria

All 1–59 months old children hospitalized to the SC in suhul general hospital both from IDP and host communities from January 01, 2023 to June 30, 2023.

#### Exclusion criteria

Those with age of less than 1 month, children with ready-to-use therapeutic food (RUTF) intolerance and those with secondary causes of undernutrition like cerebral palsy, congenital heart disease and cleft lip and palate were omitted.

### Sampling procedure and sample size

Exhaustive sampling method was used, taking all 1–59 months old children hospitalized to suhul SC from January 01, 2023 to June 30, 2023 and our final sample was 184 children. Instead of calculating the minimum sample size, we calculated the power of the study using STATA version 15 software because we took the entire children admitted to the stabilization centre to our study. Therefore, considering the actual sample size of 184 children, type-I error of 5% and Adjusted Hazard Ratio of 0.5 (for age as independent predictor of survival among SAM children) the power of the study became 99.5%, which is much higher than the expected 80%.

### Variables of the study

#### Dependent/outcome variable

Stabilizing/curing time.

Event: stabilized and/or cured and transferred to out-patient therapeutic programme (OTP).

Censored: Death, default, and medical referrals.

### Independent variables

- Main exposure variable: Age of child.
  - Exposed: children < 6 months with SAM.
  - Non-exposed: children 6–59 months old with SAM.
- Other independent variables: Study participants’ characteristics (sex, residence, age), routine medications, supplements and co-morbidities (like malaria, Pneumonia, severe anemia).

### Operational definitions

- **Stabilizing time or length of stay (LoS)**: refers to the number of days it takes from hospitalization till a child gets stabilized from medical complications and/or cured from SAM of any kind.
- **Stabilized and transferred to OTP**: 6 to 59 months old children can continue the nutritional rehabilitation phase at OTP once they get relieved from medical complications, grade three oedema and regained appetite.
- **Discharged after full recovery (Cured)**: when a child (mainly those less than six months old) fulfilled the discharge criteria as cured. The same anthropometric indicator was used to admit as well as to declare recovery and discharge from treatment. all less than six month children discharged cured were transferred to OTP for further follow- up both for the mother and other IYCF-E services.
- **Defaulted or left against medical advice (LAMA)**: those who are not found in SC for two successive days, or who leave the ward against professional advice while the child is not stabilized.
- **Death**: child who dies while receiving treatment in SC.

### Data collection tools and data quality control

#### Data collection tools

Structured questionnaire was created referring to the Ethiopian SAM management guideline. The questionnaire was put together with the patient file and necessary patient data was filled as in the relevant documents like SAM registration logbook, SAM monitoring multi-chart and patient clinical files.

### Data quality assurance

The structured checklist was prepared in English, same language used by the FMoH SAM management guideline. It was pretested in 19 patients from another SC (10% of our total sample), was revised for sequence and layout. After taking one-day training on data collection, the nutrition supervisor and nutrition nurses were responsible to complete the checklist along with their routine activity. To minimize an information bias, a prospective follow-up study was applied, data collectors strictly followed the admitted patients throughout the data collection period, the measurement of the outcome was assessed objectively (stabilized and/or cured as event and other treatment outcomes as censored) and the time of occurrence of the events were recorded timely and appropriately. The collected data was checked by the nutrition supervisor for its accuracy, completeness & consistency and corrective measures were taken on spot.

### Data processing and analysis

The collected data got coded and entered to EpiData version 4.2.0 and then exported to statistical package for social sciences (SPSS) version 25 for analysis. Before analysis, data was cleaned by checking the levels of missing values and existence of influential extreme values among independent variables and did not found any outlier. Collinearity diagnostics among independent variables was assessed using variance inflation factor (VIF) and tolerance, it was suggestive of correlation between hyperthermia and malaria with VIF of 4.376 while anemia correlated with blood transfusion with VIF and tolerance coefficients of 5.822 and 0.172 respectively. As a result, hyperthermia and anemia were dropped during modelling.

Exploratory data analysis is performed and presented in tables and percentages along with the appropriate central tendency measure (median survival time in days). Kaplan-Meier & Cox regression was applied to determine the association of independent variables with dependent. Stabilizing time from SAM was estimated using Kaplan-Meier procedure with Log Rank (Mantel-Cox) test to examine whether the observed difference of stabilizing time between different groups of predictor variables is significant or not. Chi-square test was done to determine if there were adequate cell counts for each categorical variable. Independent variables with *p-value* of < 0.15 during the bivariate cox regression were selected as candidates for multivariable analysis.

The proportionality of hazards assumption was checked by examining plots of stabilizing time for model variables. The plotted points nearly lie around a line that has unit slope and zero intercept. Overall fitness of the model was assured by Omnibus tests of model coefficients at 5% level of significance. Multivariable Cox regression was run using Forward Wald method to detect best independent predictors of stabilizing time. Finally, adjusted hazard ratio (AHR) with 95% confidence interval (CI) was used to show the strength of association and declare a statistical significance at p-value of < 0.05.

## Results

### Baseline and socio-demographic characteristics of participants

**Fig. 1:**
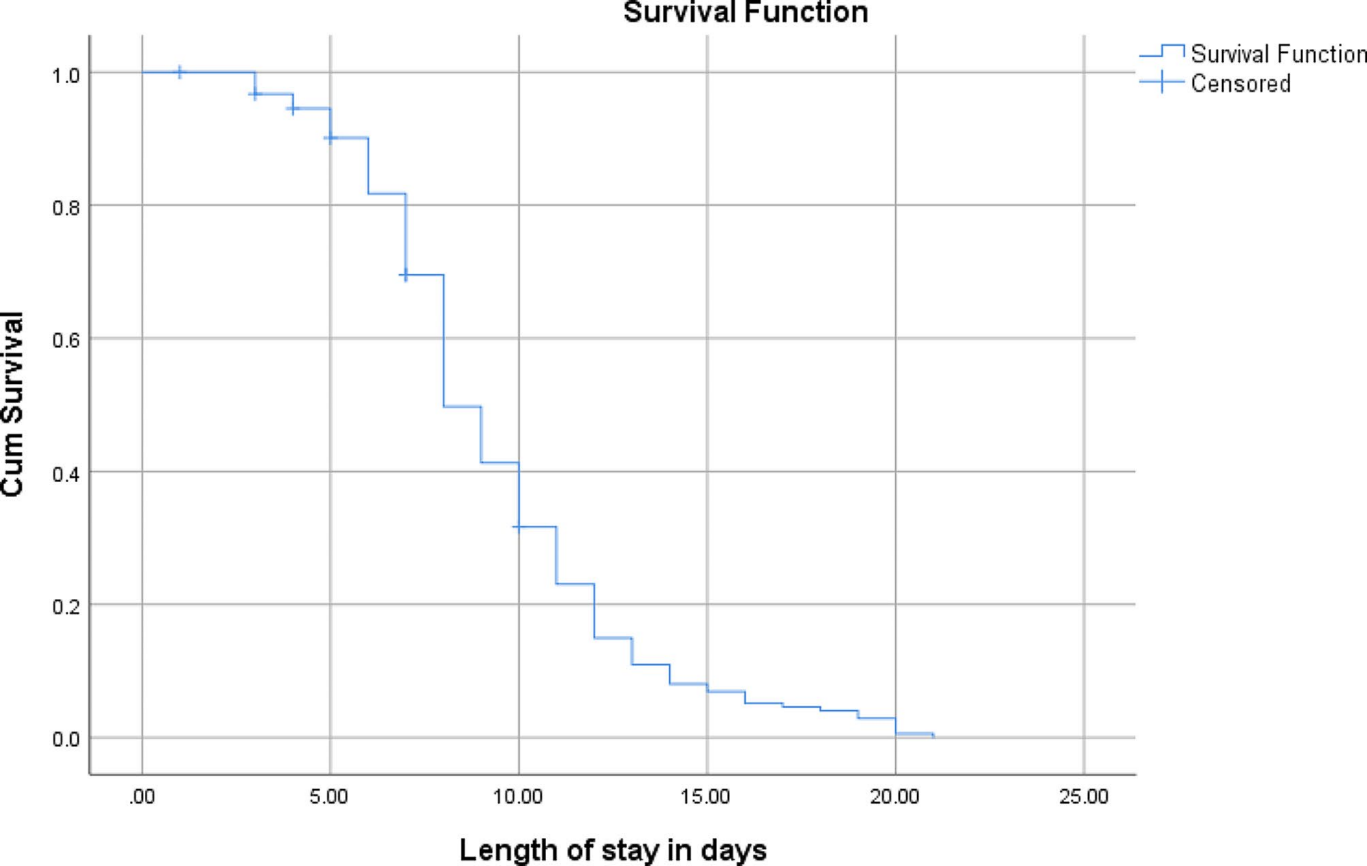
Overall survival curve of children having SAM and managed in Suhul general hospital, 2023

Out of the total 212 severely undernourished children admitted to suhul hospital, 184 were included in the study while the rest 28 were excluded due to the exclusion criteria. Comprising those 6–59 months old who got stabilized and transferred to OTP (*n* = 138, 75%) and cured infants less than six months of age (*n* = 39, 21.2%), recovery rate was 96.2% whereas defaulter and death rate found to be 2.2 and 1.6%, respectively. Weight gain was calculated for less than six months old infants because it was used as discharge criteria and their average weight gain was 12.89 g per kilogram daily. Recovery time analysis was made on the basis of demographics and other health related characteristics of study participants (Fig. 1) (Fig. 1).

### Median stabilizing time (bivariable cox-regression analysis)

**Fig. 2:**
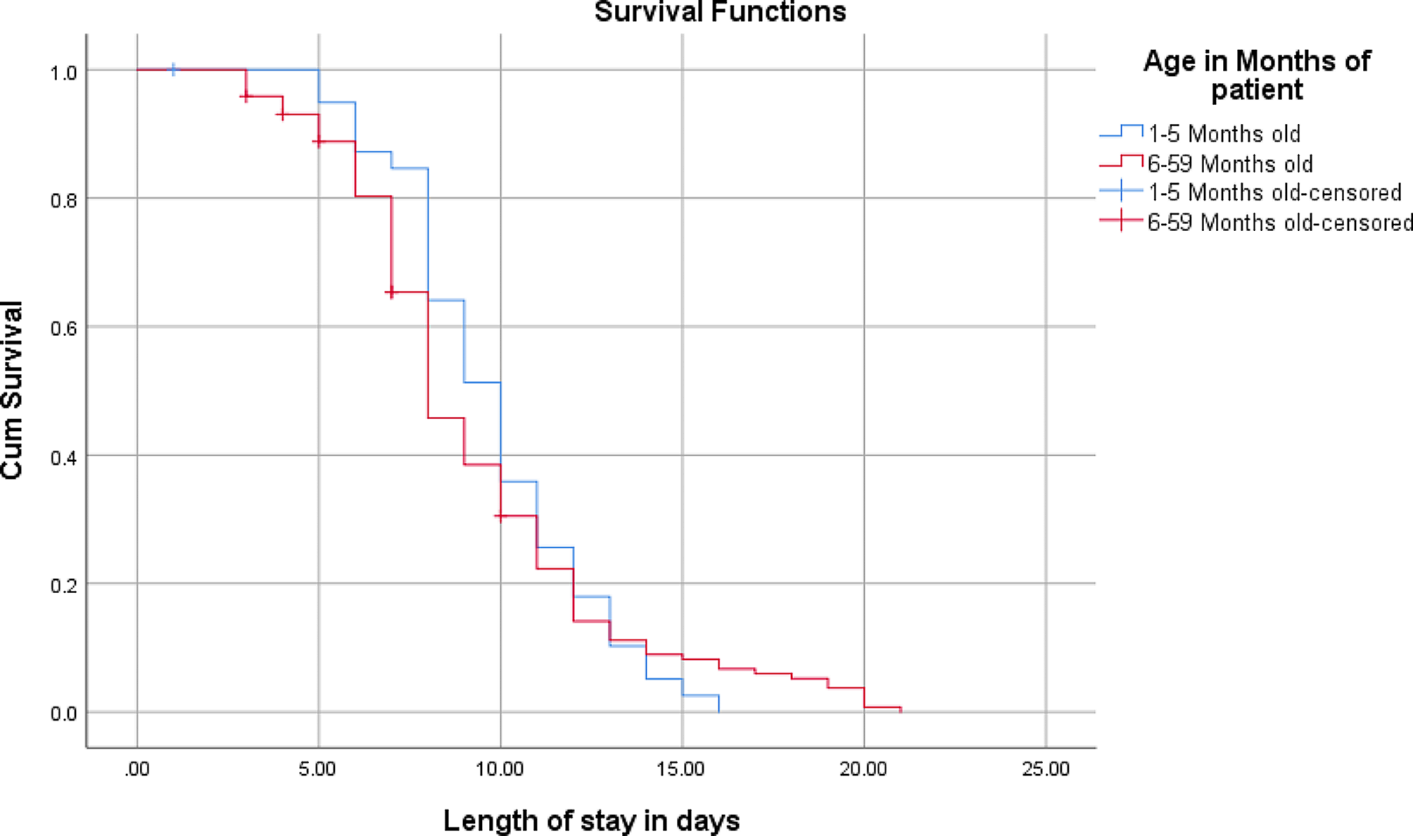
Survival function by age of children with SAM managed in Suhul general hospital, 2023

The overall median stabilizing time till outcome was 8 days (95% CI: 7.5, 8.5). The pattern of the overall survival for the whole cohort is shown below. Ninety-nine (53.8 per cent) of the children enrolled in to the study were females with the corresponding median stabilizing time of 8 days (95% CI: 7.4, 8.6) which is similar with the overall median stabilizing time. Majority (75.5%) of them are from host community both rural and urban. Mean and median age of participants was 17.9 and 16 months, respectively. Children categorized under the age group of 12–23 months had higher number of participants while those under the age category of 6–11 months had the lowest with the same estimated median stabilizing time of 8 days with a 95% CI. At 5% level of significance, age groups of less than six and 24–59 months had longer stabilizing time, 10 days. However, this observed difference is by chance as it did not show statistical significance (*p-value* 0.253) (Fig. 2).

Majority of the participants were new admissions and about 63.4% were breastfed. Dehydration (*n* = 51, 27.7%), diarrhea (*n* = 40, 21.7%), pneumonia (*n* = 36, 19.6%) and anorexia (*n* = 35, 19%) were commonly observed complications for hospitalization. most children (*n* = 149, 81%) were diagnosed as suffering from marasmus with median stabilizing time of 8 days (95% CI 7.4, 8.6), with statistically significant difference compared to those having oedematous type of SAM (kwashiorkor and marasmic-kwash) which is estimated to be 10 days with *p-value* of 0.048 (Table 1).

**Table 1:** Log Rank (Mantel-Cox) test of equality of survival distribution for sociodemographics, admission type, breastfeeding status and diagnosis in severely malnourished children hospitalized to Suhul hospital, 2023

Variable	Category	Percentage of outcome			Median survival (days)	95% CI		Log rank (Mantel-Cox)	
		Recovered Number (%)	Censored Number (%)	Total Number (%)		LB	UB	Chi-square	*p-value*
Age in months	1–5	39 (21.2)	1 (0.54)	40 (21.7)	10	8.9	11.1	4.084	0.253
	6–11	23 (12.5)	1 (0.54)	24 [13]	8	6.4	9.6		
	12–23	66 (35.9)	2 (1.1)	68 [37]	8	7.5	8.5		
	24–59	49 (26.6)	3 (1.62)	52 (28.3)	10	8.3	11.7		
	Overall	177 (96.2)	7 (3.8)	184 (100)	8	7.5	8.5		
Sex	Female	94 (51.1)	5 (2.7)	99 (53.8)	8	7.4	8.6	0.110	0.740
	Male	83 (45.1)	2 (1.1)	85 (46.2)	9	8	10		
	Overall	177 (96.2)	7 (3.8)	184 (100)	8	7.5	8.5		
Residence	Host-rural	101 (54.9)	7 (3.8)	108 (58.7)	8	7.6	8.4	1.315	0.518
	IDP	45 (24.5)	0 (0)	45 (24.5)	10	8.2	11.8		
	Host-urban	31 (16.8)	0 (0)	31 (16.8)	9	7.9	10.1		
	Overall	177 (96.2)	7(3.8)	184 (100)	8	7.5	8.5		
Type of admission	New	173 (94)	7 (3.8)	180 (97.8)	9	8.5	9.5	0.326	0.568
	Re-admission	4 (2.2)	0 (0)	4 (2.2)	8	6.3	9.7		
	Overall	177 (96.2)	7 (3.8)	184 (100)	8	7.5	8.5		
Breastfeeding	Yes	113 (61.4)	4 (2.2)	117 (63.6)	9	8.4	9.6	3.373	0.185
	Not indicated	36 (19.6)	1 (0.5)	37 (20.1)	10	7.8	12.2		
	No	28 (15.2)	2 (1.1)	30 (16.3)	8	7.6	8.4		
	Overall	177 (96.2)	7 (3.8)	184 (100)	8	7.5	8.5		
Diagnosis	Marasmus	144 (78.3)	5 (2.7)	149 (81)	8	7.4	8.6	6.070	0.048
	Kwashiorkor	23 (12.5)	2 (1.1)	25 (13.6)	10	8.2	11.8		
	Marasmic-Kwash	10 (5.4)	0 (0)	10 (5.4)	10	5.4	14.6		
	Overall	177 (96.2)	7 (3.8)	184 (100)	8	7.5	8.5		

### Treatment, routine medications and feeding

One hundred and seventy-two (93.5%) of the study participants received intravenous antibiotics and the remaining took oral medications. sixty-two (33.7%) children fed via nasogastric tube while 14 (7.6%) children transfused blood. Though all eligible children were dewormed, only seventy-eight (42.4%) out of the eligible 138 were vaccinated for measles (Table 2).

**Table 2:** Log Rank (Mantel-Cox) test of equality of survival distribution for treatment, routine medication and feeding for children with SAM managed in Suhul hospital, 2023

Variable	Category	Percentage of outcome			Median survival (days)	95% CI		Log rank (Mantel-Cox)	
		Recovered no. (%)	Censored no. (%)	Total no. (%)		LB	UB	Chi-square	p-value
Appetite test	Failed	32 (17.4)	3 (1.6)	35 [19]	7	6.6	7.4	48.85	< 0.001
	Not indicated	145 (78.8)	4 (2.2)	149 (81)	9	8.2	9.8		
	Overall	177 (96.2)	7 (3.8)	184 (100)	8	7.5	8.5		
Pneumonia	Yes	36 (19.6)	0 (0)	36 (19.6)	8	7.5	8.5	1.023	0.312
	No	141 (76.6)	7 (3.8)	148 (80.4)	9	8.4	9.6		
	Overall	177 (96.2)	7 (3.8)	184 (100)	8	7.5	8.5		
Diarrhea	Yes	40 (21.7)	0 (0)	40 (21.7)	10	9.1	10.9	0.965	0.326
	No	137 (74.5)	7 (3.8)	144 (78.3)	8	7.6	8.4		
	Overall	177 (96.2)	7 (3.8)	184 (100)	8	7.5	8.5		
Vomiting	Yes	6 (3.3)	0 (0)	6 (3.3)	9	7.4	10.6	1.477	0.224
	No	171 (92.9)	7 (3.8)	178 (96.7)	8	7.5	8.5		
	Overall	177 (96.2)	7 (3.8)	184 (100)	8	7.5	8.5		
Malaria	Positive	20 (10.9)	1 (0.5)	21 (11.4)	12	7.7	16.3	16.176	< 0.001
	Negative	31 (16.8)	2 (1.1)	33 (17.9)	8	6.6	9.4		
	Not indicated	126 (68.5)	4 (2.2)	130 (70.7)	8	7.4	8.6		
	Overall	177 (96.2)	7 (3.8)	184 (100)	8	7.5	8.5		
Lethargy/unconscious	Yes	6 (3.3)	1 (0.5)	7 (3.8)	17	8.6	25.4	10.927	0.001
	No	171 (92.9)	6 (3.3)	177 (96.2)	8	7.5	8.5		
	Overall	177 (96.2)	7 (3.8)	184 (100)	8	7.5	8.5		
Skin lesions	Yes	15 (8.2)	1 (0.5)	16 (8.7)	10	7.5	12.5	6.557	0.01
	No	162 (88)	6 (3.3)	168 (91.3)	8	7.5	8.5		
	Overall	177 (96.2)	7 (3.8)	184 (100)	8	7.5	8.5		
Level of dehydration	No dehydration	127 (69)	6 (3.3)	133 (72.3)	8	7.6	8.4	18.670	< 0.001
	Moderate/Some	39 (21.2)	0 (0)	39 (21.2)	10	8.9	11.1		
	Severe/shock	11 [6]	1 (0.5)	12 (6.5)	13	9	17		
	Overall	177 (96.2)	7 (3.8)	184 (100)	8	7.5	8.5		
Blood transfusion	Yes	13 (7.1)	1 (0.5)	14 (7.6)	17	10.1	23.9	29.199	< 0.001
	No	164 (89.1)	6 (3.3)	170 (92.4)	8	7.5	8.5		
	Overall	177 (96.2)	7 (3.8)	184 (100)	8	7.5	8.5		
IV fluid	Yes	13 (7.1)	1 (0.5)	14 (7.6)	12	9.7	14.3	7.580	0.006
	No	164 (89.1)	6 (3.3)	170 (92.4)	8	7.5	8.5		
	Overall	177 (96.2)	7 (3.8)	184 (100)	8	7.5	8.5		
IV Antibiotics	Yes	167 (90.8)	5 (2.7)	172 (93.5)	9	8.5	9.5	21.092	< 0.001
	Not indicated	10 (5.4)	2 (1.1)	12 (6.5)	6	5.1	6.9		
	Overall	177 (96.2)	7 (3.8)	184 (100)	8	7.5	8.5		
NG tube feeding	Yes	58 (31.5)	4 (2.2)	62 (33.7)	11	10	12	17.683	< 0.001
	Not indicated	119 (64.7)	3 (1.6)	122 (663)	8	7.6	8.4		
	Overall	177 (96.2)	7 (3.8)	184 (100)	8	7.5	8.5		
Measles vaccination	Yes	78 (42.4)	0 (0)	78 (42.4)	8	7.5	8.5	2.796	0.247
	No	56 (30.4)	4 (2.2)	60 (32.6)	8	7	9		
	Not indicated	43 (23.4)	3 (1.6)	46 [25]	9	7.8	10.2		
	Overall	177 (96.2)	7 (3.8)	184 (100)	8	7.5	8.5		
Deworming	Yes	62 (33.7)	0 (0)	62 (33.7)	10	8.7	11.3	4.512	0.034
	Not indicated	115 (62.5)	7 (3.8)	122 (66.3)	8	7.5	8.5		
	Overall	177 (96.2)	7 (3.8)	184 (100)	8	7.5	8.5		

### Predictors of stabilizing time

Twenty independent variables were analysed and variables with *p-value* < 0.15 during Bivariate analysis were taken to multivariable analysis using Cox proportional hazard regression. After discovering validity of the model assumptions and adjustment, five independent significant predictors of stabilizing time were identified, which are; appetite test, blood transfusion, IV fluid, Antibiotics and nasogastric tube (NGT) feeding (Table 3.)

**Table 3:** Predictors of stabilizing time for children with SAM managed in Suhul general hospital, 2023

Variable	Category	Percentage of outcome			CHR (95% CI)	AHR (95% CI)	*p-value*
		Recovered	Censored	Total			
		Number (%)	Number (%)	Number (%)			
Diagnosis	Marasmus	144 (78.3)	5 (2.7)	149 (81)	1	1	0.614
	Kwashiorkor	23 (12.5)	2 (1.1)	25 (13.6)	0.640 (0.407–1.005)	0.726 (0.384–1.371)	
	Marasmic-Kwash	10 (5.4)	0 (0)	10 (5.4)	0.682 (0.358-1.300)	0.915 (0.444–1.888)	
Appetite test	Failed	32 (17.4)	3 (1.6)	35 [19]	1	1	< 0.001*
	Not indicated	145 (78.8)	4 (2.2)	149 (81)	0.275 (0.181–0.416)	0.338 (0.221–0.518)	
Malaria	Positive	20 (10.9)	1 (0.5)	21 (11.4)	1	1	0.280
	Negative	31 (16.8)	2 (1.1)	33 (17.9)	2.285 (1.217–4.290)	1.530 (0.718–3.258)	
	Not indicated	126 (68.5)	4 (2.2)	130 (70.7)	2.269 (1.543–4.719)	1.105 (0.535–2.284)	
Lethargic	Yes	6 (3.3)	1 (0.5)	7 (3.8)	1	1	0. 965
	No	171 (92.9)	6 (3.3)	177 (96.2)	3.199 (1.390–7.362)	0.977 (0.343–2.784)	
Skin lesions	Yes	15 (8.2)	1 (0.5)	16 (8.7)	1	1	0.543
	No	162 (88)	6 (3.3)	168 (91.3)	1.866 (1.074–3.240)	1.267 (0.591–2.715)	
Level of dehydration	No dehydration	127 (69)	6 (3.3)	133 (72.3)	1	1	0.261
	Moderate/Some	39 (21.2)	0 (0)	39 (21.2)	0.675 (0.469–0.973)	0.880 (0.585–1.325)	
	Severe/shock	11 [6]	1 (0.5)	12 (6.5)	0.342 (0.182–0.642)	0.369 (0.111–1.228)	
Blood transfusion	Yes	13 (7.1)	1 (0.5)	14 (7.6)	1	1	< 0.001*
	No	164 (89.1)	6 (3.3)	170 (92.4)	5.537 (2.599–11.798)	5.825 (2.568–13.211)	
IV fluid	Yes	13 (7.1)	1 (0.5)	14 (7.6)	1	1	0.025*
	No	164 (89.1)	6 (3.3)	170 (92.4)	2.003 (1.131–3.549)	2.017 (1.094–3.717)	
IV antibiotics	Yes	167 (90.8)	5 (2.7)	172 (93.5)	1	1	0.016*
	Not indicated	10 (5.4)	2 (1.1)	12 (6.5)	3.781 (1.964–7.279)	2.288 (1.164-4.500)	
NG tube feeding	Yes	58 (31.5)	4 (2.2)	62 (33.7)	1	1	0.02*
	Not indicated	119 (64.7)	3 (1.6)	122 (663)	1.829 (1.327–2.521)	1.485 (1.065–2.071)	
Deworming	Yes	62 (33.7)	0 (0)	62 (33.7)	1	1	0.227
	Not indicated	115 (62.5)	7 (3.8)	122 (66.3)	1.356 (0.987–1.863)	0.770 (0.504–1.177)	

Bold values indicate significance at p-value < 0.05

NB: * = significant predictors of stabilizing time

Patients for whom appetite test was not indicated (rather hospitalized with other medical complications or age < 6 month) were 66.2% (AHR = 0.338; 95% CI: 0.221–0.518) less likely to stabilize quickly from SAM compared to children admitted due to failed appetite. Children who were not blood transfused as well as those who were not resuscitated with IV fluid found to be 5.8 (AHR = 5.825; 95% CI: 2.568–13.211) and 2 (AHR = 2.017; 95% CI: 1.094–3.717) folds more likely to get stabilized/cured early in reference to those transfused and received IV fluids respectively. Likewise, children received oral antibiotics and those who consumed the therapeutic milk orally were 2.3 (AHR = 2.288; 95% CI: 1.164-4.500) and 1.5 (AHR = 1.485; 95% CI: 1.065–2.071) times better in earlier recovery compared to their counterparts who took IV antibiotics and fed via NG tube respectively.

## Discussion

Most study participants had a diagnosis of marasmus (severe wasting) at admission, similar with current findings in other parts of the country [24, 25, 33, 34]. During Kapan-Meier estimation of survival, we found statistically significant difference in stabilizing time with diagnosis or type of SAM. This is the same with the research finding from Asosa general hospital [30], but contrary to the findings from public hospitals in Aksum [27] and south wollo zone of Amhara regional state [26]. The overall median stabilizing time was 8 days, which is exactly the same estimate with current finding from Sidama region [35]. However, this LoS is quite shorter in comparison to reports from studies conducted in three hospitals in Addis Ababa [36] as well as three hospitals from Oromia regional state [37] who declared recovery time of 18.6 and 21 days respectively. This difference might be attributed to lack of adherence to the management protocol and lack of supplies between the hospitals.

By discovering a recovery rate of 96.2 per cent with 2.2 and 1.6 per cent of defaulter and death rate respectively, this fulfilled the standards set by the SPHERE association during humanitarian interventions [38]. This is far better than recent similar researcher’s reports from Addis Ababa Yekatit 12 hospital [39] and Pawe general hospital in Benishangul region [29], who stated the final findings did not meet the SPHERE performance indicators. This may be as a result of the inclusion of children less than six months old in our research, who received treatment until they recovered, as well as the assistance of numerous humanitarian organizations for services connected to IYCF and health promotion. However, our results are consistent with the recently published data in an area where there was similar humanitarian crisis in Nigeria [37], where 86% of participants had marasmus, 95.7% of them recovered, and the median stabilization period was six days [40].

The median stabilizing time of this study was found to be significantly shorter (better) than similar studies conducted in different regions of the country before the humanitarian crisis [24, 26]. The reason behind the difference of the findings before and during the humanitarian crisis could be due to the involvement of Non-Governmental Humanitarian Organizations in the study area because there was high involvement of NGOs in Suhul Hospital after the peace agreement to support the overall administrative, clinical, financial support and food assistance to patients, clients and hospital staff. This finding could be in contrary to other settings of the region, which did not have enough support by different actors during the crisis.

Adjusting for other variables during multivariable Cox regression, appetite test was independent predictor of stabilizing time, which is consistent with study in Adama hospital medical college [41] and another meta-analysis [42]. We found treatments like blood transfusion, IV fluid resuscitation and receiving IV antibiotics as significant predictors of stabilizing time. Our findings are in line with reports from southern Ethiopia [21], Uganda [43] and Malawi [44]. Likewise, children who were able to take therapeutic milk orally had 1.5 times better chance of stabilizing unlike those who were fed with NG tube. Scholars from east and northeast parts of Ethiopia also found NG tube feeding as an independent predictor of SAM management in SC [33, 34].

## Conclusion and recommendations

The median stabilization time of children admitted to SC of Suhul Hospital is consistent with the SPHERE association set of standards and way better than most of recent studies in the subject area.

Admission of children using failure for appetite test, and provision of special treatments like blood transfusion, IV fluid administration, IV antibiotics and feeding via NG tube were identified as significant predictors of stabilizing time. Therefore, Suhul Hospital and concerned humanitarian actors should focus on capacitating the technical staff mainly on management of complications like severe anemia, shock and NG tube insertion. Additional attention should be paid for maintenance and sustainability issues of the blood bank as well as the laboratory. Involvement of Non-Governmental Humanitarian Organizations to prevent mortality of children admitted to health facilities during and after a humanitarian crisis is crucial. Further pre-post experimental studies which compare the stabilizing time before and after crisis are recommended.

## Data Availability

All materials and analyzed data are included in the manuscript. However, when additional anonymized data is requested, it can be given from the corresponding author.

## Abbreviations

AHR: Adjusted Hazard Ratio
CHR: Crude Hazard Ratio
CI: Confidence Interval
FMoH: Federal Ministry of Health
IDP: Internally Displaced Person
IYCF: Infant and Young Child Feeding
LoS: Length of Stay
NGO: Non-Governmental Organization
NGT: Naso-Gastric Tube
SAM: Severe Acute Malnutrition
SC: Stabilization Center
SPSS: Statistical Package for Social Sciences (Statistical Product and Service Solutions)
UNICEF: United Nations International Children’s Emergency Fund
